# Broad ligament cystic lymphangioma: A case report

**DOI:** 10.1186/1752-1947-2-310

**Published:** 2008-09-23

**Authors:** K Harish, SR Karthik, CS Manjunath

**Affiliations:** 1 Department of Surgical Oncology, MS Ramaiah Medical College and Hospital, Bangalore 560054, India; 2 Bangalore Institute of Oncology, Raja Ram Mohan Roy Extension, Bangalore 560027, India; 3 Department of Oncology, MS Ramaiah Medical College and Hospital, Bangalore 560054, India; 4 Gokula Metropolis Lab, MS Ramaiah Memorial Hospital, Bangalore 560054, India

## Abstract

**Introduction:**

Cystic lymphangiomas are uncommon tumors that can arise from any part of the body. They can pose a diagnostic and therapeutic challenge. They are more common in infants and children than adults. Broad ligament cystic lymphangioma is extremely rare.

**Case presentation:**

A 70-year-old multiparous woman presented with an abdominal mass of 20-year duration. A large cystic swelling was detected on computed tomography scan that was found to arise from the left adnexal region. This 19 kg lesion was found arising from the broad ligament. It was successfully removed. A detailed pathological study, including immunohistochemistry, was required to diagnose the lesion as a cystic lymphangioma.

**Conclusion:**

Lymphangiomas should be treated with total surgical excision. Broad ligament lymphangiomas are extremely rare but must be considered as a differential diagnosis of cystic lesions in that region.

## Introduction

Cystic lymphangiomas are common in infants and children, but adult cystic lymphangiomas are rare. Although they can occur at any site in the body, cystic lymphangiomas of the broad ligament are extremely rare [1].

## Case presentation

**Figure 1:**
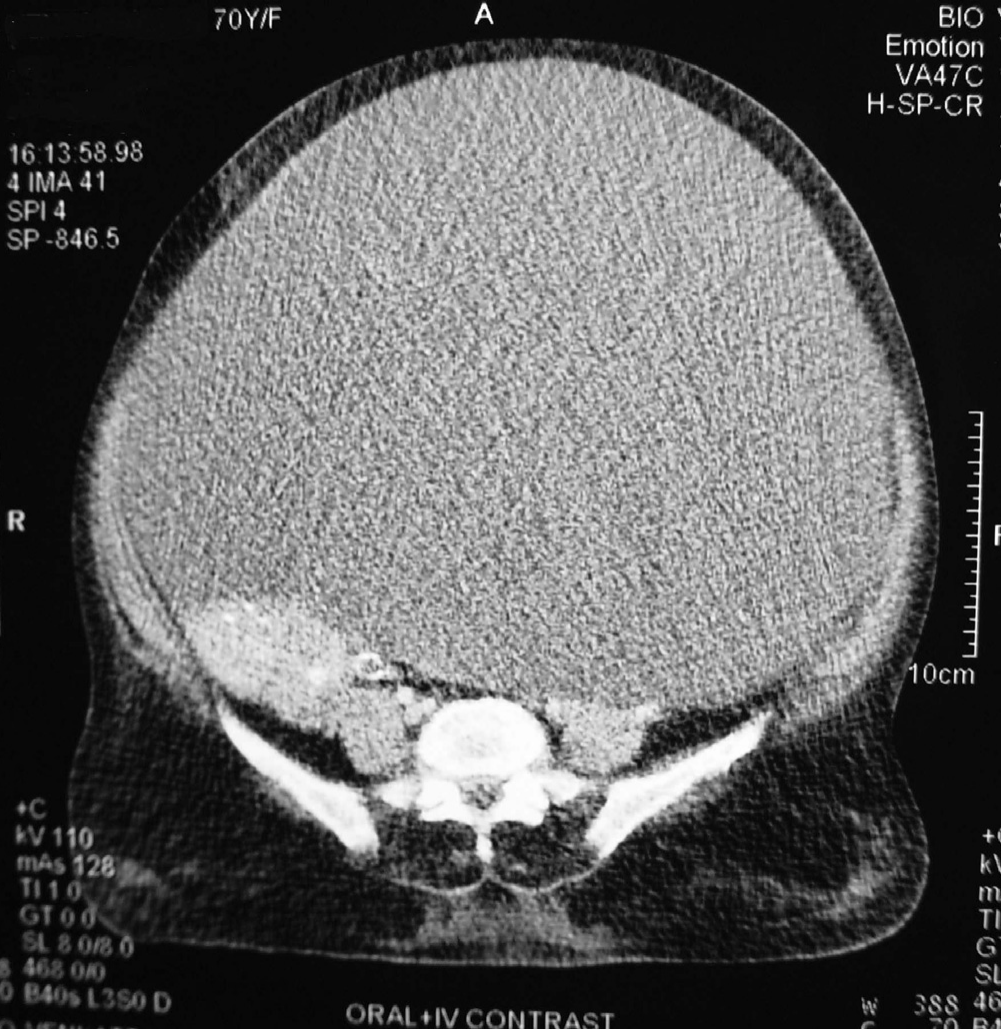
Computed tomography scan of the abdomen and pelvis showing a large cystic mass occupying the entire abdomen and pelvis.

**Figure 2:**
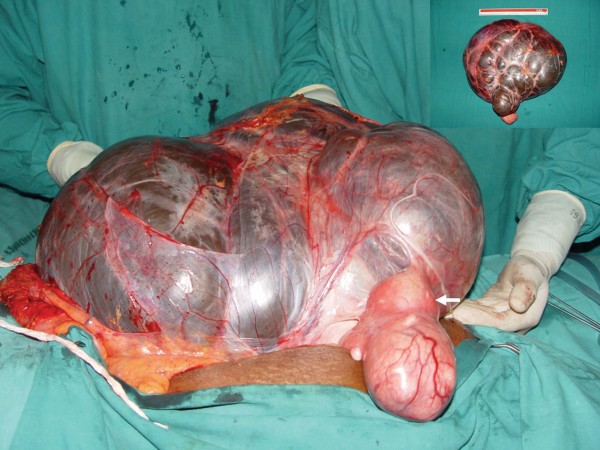
**Excision of the tumor**. Note that the origin is from the left broad ligament. The uterus is indicated with a bold white arrow. The inset shows the entire tumor, measuring 47 cm across.

A 70-year-old multiparous woman presented with a huge abdominal swelling of 20-year duration. The patient had been unable to walk for 3 months. A computed tomography (CT) scan revealed a huge cystic swelling in the abdomen, possibly arising from the left adnexal region (Figure 1). After presurgical workup, the patient underwent an exploratory laparotomy. A large cystic mass was found occupying the entire abdomen. The lower limit of the mass was in close relation and adherent to the uterus on its left side. The left ovary and fallopian tube were not separately visualized. The surgery performed included pan-hysterectomy and right salpingo-oophorectomy along with the excision of the cystic mass (Figure 2). The tumor weighed 19 kg. The postoperative period was uneventful.

**Figure 3:**
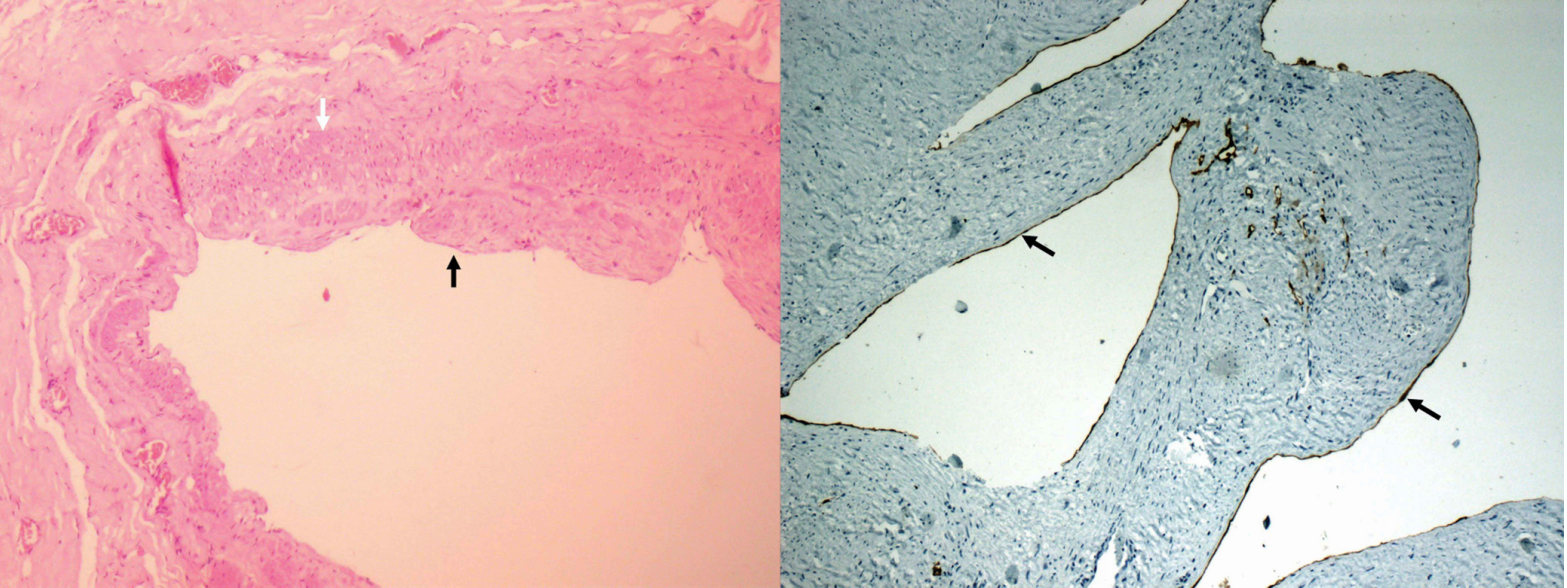
**Histology and immunohistochemistry**. The left panel shows a microphotograph (magnification ×400, hematoxylin and eosin stain) showing smooth muscle in the wall of the cyst, indicated by a white bold arrow, and flattened endothelial-like cells lining the cyst wall, indicated by a bold black arrow. The right panel shows immunohistochemistry by marker D2-40 identifying the lymphatic endothelium, indicated by bold black arrows.

Pathological gross findings were those of a very large multiseptate cystic lesion covered with serosa. Microscopy revealed that the cyst wall had bundles of smooth muscle with connective tissue. In addition, the cyst wall was lined internally with epithelium (Figure 3). This tumor, arising from the left broad ligament, was found to be benign. Although the uterus showed multiple leiomyomata, in view of the epithelial lining, cystic degeneration of leiomyoma was considered unlikely. On immunohistochemical study, the tumor cyst wall stained positively with the lymphatic marker D2-40 (Figure 3, right panel); hence, the tumor was diagnosed as cystic lymphangioma. The patient has had regular follow-up for 2 years, and there have been no signs of recurrence.

## Discussion

Lymphangiomas are benign tumors of the lymphatic system. They are classified as cavernous, lymphangioma simplex, or cystic lymphangioma [2]. Cystic lymphangioma, first described in 1828 by Redenbacker, is a malformation of the lymphatic system. It can affect any site in the body but is seen more commonly in the head and neck region and the axilla. It is also reported to occur in the mediastinum, retroperitoneum, and other regions [2,3]. Cystic lymphangiomas most commonly affect children. About 90% of these lymphangiomas manifest before 2 years of age and are very rarely encountered in adults [3]. The reported patient was aged 70 years at presentation.

Lymphangiomas in children are considered to arise from sequestered lymphatic sacs that fail to communicate with the draining lymphatic channels. This is a widely accepted theory. However, the etiology in the adult population is controversial. Some authors believe that the adult manifestations are a result of delayed proliferation of congenital or acquired lymphoid nests after stimuli such as respiratory infection or local trauma [4]. Others dispute the congenital origin and propose that adult cystic lymphangiomas arise as a result of trauma alone [5]. There was no history of trauma in this patient.

Radiographic evaluation with magnetic resonance imaging or CT is invaluable for the diagnosis and determination of the extent of the lesion. In addition, it is essential in defining normal anatomical structures that need to be preserved when surgical excision is performed [4]. Accurate pre-operative diagnosis of cystic lymphangioma is uncommon [6] and was a problem faced in the reported case. The CT scan revealed a huge cystic lesion, but identification of the site of origin and diagnosis were not possible.

Lymphangiomas are treated by surgical excision. Complete excision of the mass with negative surgical margins is the optimal treatment, and the results are excellent [7]. Intra-abdominal lymphangiomas have a 10% postoperative recurrence rate for incompletely excised lesions. We were able to achieve total excision of the cyst.

Effective immunohistochemical markers specific for lymphatic endothelial cells have been reported including lymphatic vessel endothelial receptor 1, vascular endothelial growth factor receptor 3, and Prox-1. However, the antibodies against these markers are available only for frozen section specimens. More recently, a new monoclonal antibody, D2-40, has become available; this is a specific marker of lymphatic endothelium, since it does not stain vascular endothelium [8]. In this case, diagnosis was made only after immunostaining with the lymphatic marker D2-40.

We could only find one case of broad ligament cystic lymphangioma reported in the literature [1]. The case presented here is probably only the second case of broad ligament lymphangioma to be reported. The lesion weighed 19 kg and is probably one of the largest to be reported. Malignant transformations of cysts are rare and have been reported only once [9]. Such a transformation is an exception rather than a rule.

## Conclusion

Adult cystic lymphangiomas of the broad ligament are very rare benign tumors. Total surgical removal is the treatment of choice. This is a report of one such case diagnosed with the help of the lymphatic marker D2-40, treated successfully with surgery, and recurrence-free 2 years later. Although rare, cystic lymphangiomas must be considered in the differential diagnosis of cystic lesions in the abdomen and pelvis.

## Abbreviations

CT: computed tomography.

## Competing interests

The authors declare that they have no competing interests.

## Authors' contributions

KH contributed to the conception, design, gathering data, and revision of the manuscript draft. SRK contributed to obtaining the data and drafting the manuscript. CSM contributed to obtaining the data, the pathological review, and drafted part of the manuscript. All authors read and approved the final manuscript.

## Consent

Written informed consent was obtained from the patient for publication of this case report and any accompanying images. A copy of the written consent is available for review by the Editor-in-Chief of this journal.
